# Anxiety of pandemic and distance learning as predictor of decrease in satisfaction, competence and engagement in students during digital education period

**DOI:** 10.1192/j.eurpsy.2022.867

**Published:** 2022-09-01

**Authors:** G. Soldatova, E. Rasskazova

**Affiliations:** 1 Lomonosov Moscow State University, Faculty Of Psychology, Moscow, Russian Federation; 2 Moscow State University, Clinical Psychology, Moscow, Russian Federation

**Keywords:** Anxiety of pandemic, distance learning, digital education

## Abstract

**Introduction:**

Transition of educational institutions to distance learning in pandemic was found to be associated with students’ complaints about difficulties, decrease in effectiveness, interest and well-being (Herbert et al., 2021, Almomani et al., 2021, Puljak et al., 2020).

**Objectives:**

The aim was to reveal psychological predictors of decreased academic satisfaction, competence, and engagement among students in the digitalization of education during a pandemic.

**Methods:**

In December 2020 220 students 18-33 years old were asked about their learning difficulties, academic satisfaction, competence and engagement before the pandemic and now (Cronbach’s alphas .66-.90), well-being (Diener et al., 1985, Diener et al., 2010), educational motivation (Sheldon et al., 2017), pandemic anxiety (Tkhostov, Rasskazova, 2020).

**Results:**

There was a decrease in academic satisfaction, subjective competence and engagement, with moderate learning difficulties (F=60.4-63,3, p<.01, η²=.22). More pronounced learning difficulties during a pandemic were found in students with higher level of negative emotions, lower integrated learning motivation, higher anxiety due to the transition to distance learning and due to a violation of security online (p<.01). The decrease in academic satisfaction, competence and engagement were maximal among students with a lower level of positive emotions, higher level of amotivation, anxiety due to the transition to distance learning and violation of privacy and security online (p<.01).

**Conclusions:**

Students with higher negative emotions and distance learning anxiety regardless of their skills were more vulnerable to the changes in learning in pandemic. The study was funded by Russian Science Foundation project № 18-18-00365.

**Disclosure:**

The study was funded by Russian Science Foundation project № 18-18-00365.

